# Pt-Co Alloys-Loaded Cubic SiC Electrode with Improved Photoelectrocatalysis Property

**DOI:** 10.3390/ma10080955

**Published:** 2017-08-16

**Authors:** Dan Liu, Tao Yang, Junhong Chen, Kuo-Chih Chou, Xinmei Hou

**Affiliations:** 1 State Key Laboratory of Advanced Metallurgy, University of Science and Technology Beijing, Beijing 100083, China; zhangliqin_ustb@163.com (D.L.); yt069014241@163.com (T.Y.); kcc126@126.com (K.-C.C.); 2 School of Materials Science and Engineering, University of Science and Technology Beijing, Beijing 100083, China; cjh2666@126.com

**Keywords:** photoelectrocatalytic, 3C-SiC, Pt-Co alloys, nanowires, powders

## Abstract

A novel composite photocatalyst was synthesized by loading 5 wt % of platinum cobalt alloy on 3C-SiC nanowires and powder (Pt-Co-SiC) respectively via a simple polyol reduction method. Pt-Co-SiC were comprehensively characterized by SEM, HRTEM, XRD, PL, and XPS. The results indicated that Pt-Co nanoparticles in the size of 2–5 nm were dispersed homogeneously in the SiC nanowires and powders. The photocurrent response of the Pt-Co-SiC increased remarkably with increasing Pt content and the best performance was observed with the sample of Pt_3_Co-SiC. Especially, the Pt_3_Co-SiC nanowires photoelectrode exhibited improved cathodic current density (0.14 mA·cm^−2^) under the simulated sunlight, which was about 10 times higher than the Pt_3_Co-SiC powders. The H_2_ production rate for the Pt_3_Co-SiC nanowires is 30 times more than that of the pure SiC nanowires. The enhancement of the Pt-Co-SiC properties could be ascribed to the fact that more visible light was harvested and the photogenerated electron and the interfacial electron transfered more easily.

## 1. Introduction

Photocatalytic and photoelectrochemical hydrogen evolution from water using semiconductor-based materials has attracted considerable attention since Fujishima’s discovery of photoelectrocatalytic H_2_ evolution over TiO_2_ in 1972 [1,2,3,4,5,6,7]. In recent years, cubic silicon carbide (3C-SiC) has been widely investigated as a promising environment-friendly semiconductor photocatalyst for hydrogen evolution from water because of its high chemical stability, strong thermostability, and appropriate band gap (2.4 eV for cubic SiC) [8,9,10,11,12,13]. However, 3C-SiC suffers from a similar problem as other semiconductors, that is, rapid recombination of photogenerated electron-hole pairs as well as low surface activation ability [14,15]. To overcome the limitations, various metals have been adopted as co-catalysts supported on the surface of the semiconductor, serving as highly active sites for water decomposition for the improvement of the charge separation of semiconductor [16,17].

Among the metals, low-cost non-noble metal co-catalysts including metal sulfides [18], transition metals [19], and transition metal-based complexes [20] have been investigated as alternative co-catalysts for water splitting besides noble metals [21]. Although some progress has been made in recent years, the non-noble metal co-catalysts still exhibit lower catalytic properties or require special catalytic environments than noble metals. Therefore, the noble metals, especially Pt, still play an irreplaceable role in the photocatalytic hydrogen evolution reaction, mainly because they can increase the charge separation and transfer, as well as decrease activation energy [22,23]. However, the prohibitive cost and scarcity of Pt greatly impedes its practical application. Toward this end, Pt alloys with different transition metals (cobalt, nickel, and iron) have been extensively explored. Compared with pure Pt, Pt-based alloys imply an opportunity to reduce material costs and meanwhile maintain higher photocatalytic activity [24]. Among the Pt-based alloys, platinum-cobalt (Nanowires) alloy is especially popular because of its small size, excellent self-stability, and high catalytic activity [25,26,27].

Based on the above mentioned facts, the adoption of Nanowires to modify SiC is expected, with the aim of effectively improving the separation efficiency of the electron-hole pairs and catalytic activity. In this study, Pt-Co-SiC is synthesized via an ethylene glycol reduction method. The advantages of the ethylene glycol reduction method are the synthesis of nanoparticles with good dispersion and uniform morphology. The photoelectrocatalytic activity was measured on the electrochemical workstation under visible light irradiation. Photocatalytic hydrogen production experiments were carried out in an air-free, closed gas circulation system whose reaction cell was made of quartz. Based on this, the possible catalytic mechanisms for the improved photocatalytic performance are proposed.

## 2. Results and Discussion

### 2.1. Characterization of SiC, Modified with the Pt-Co Alloy

**Figure 1:**
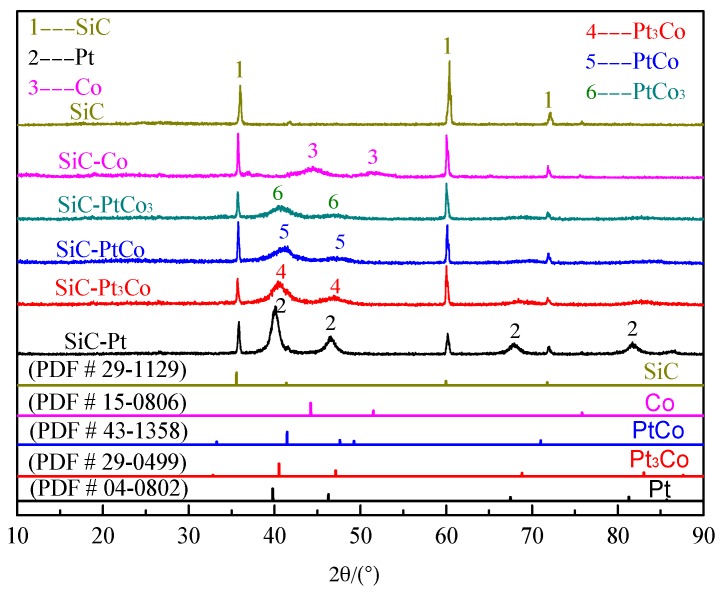
X-ray diffraction (XRD) and corresponding PDF standard patterns of the pure SiC nanowires, and SiC nanowires loaded with Pt-Co alloy.

**Figure 2:**
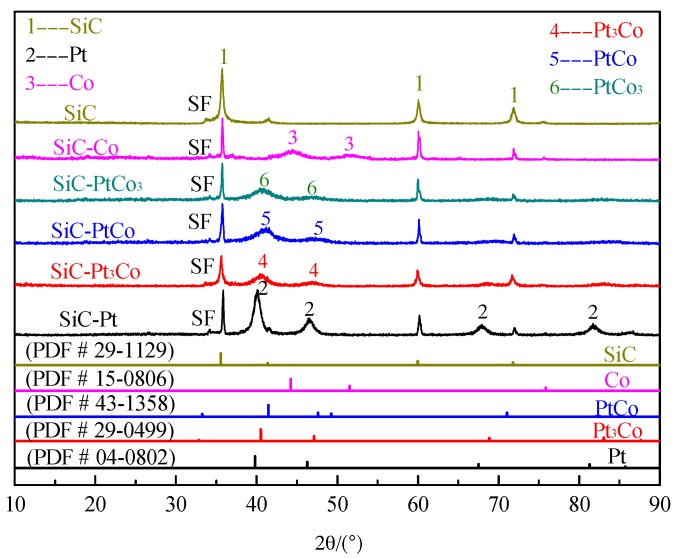
XRD and corresponding PDF standard patterns of the pure SiC powders, and SiC powders loaded with Pt-Co alloy.

The XRD patterns of the pristine and modified SiC nanowires are shown in Figure 1. The major peaks at 2θ = 35.70°, 60.10°, and 71.90° can be indexed to the (111), (220), and (311) reflections of cubic SiC (PDF # 29-1129). The peaks at 2θ of 39.7°, 46.3°, 67.5°, and 81.2° of the catalyst agree with the peaks of (111), (200), (220), and (311) of Pt (PDF # 04-0802), respectively. The diffraction peaks at 44.2° and 51.5°, which matche the reflection planes of (111) and (200), can be indexed to Co (PDF # 15-0806). Two distinct peaks at 2θ = 40.5° and 47.1° are found for the Pt_3_Co alloy. These fit the (111) and (200) planes of the Pt_3_Co alloy well (PDF # 29-0499). The peaks at 2-theta of 41.2° and 47.71° match the reflection planes of (111) and (200) in the PtCo alloy (PDF # 43-1358). These two diffraction peaks of PtCo_3_ are in good agreement with those reported in the literature [28]. Obviously, the diffraction peaks of PtCo_x_ present small movement towards a higher degree angle region when compared to pure Pt, while it is still in a lower degree angle compared with Co. The PtCo_x_ lattices contract due to the fact that diffraction peaks at higher degree angles stand for smaller crystal lattices. The contraction of PtCo_x_ lattices may be attributed to the substitution of Pt atoms with smaller Co atoms [29]. As for the pristine and modified SiC powders, the XRD patterns show the similar phenomenon as that of SiC nanowires except for stacking faults (SF) existing in SiC powder (Figure 2).

**Figure 3:**
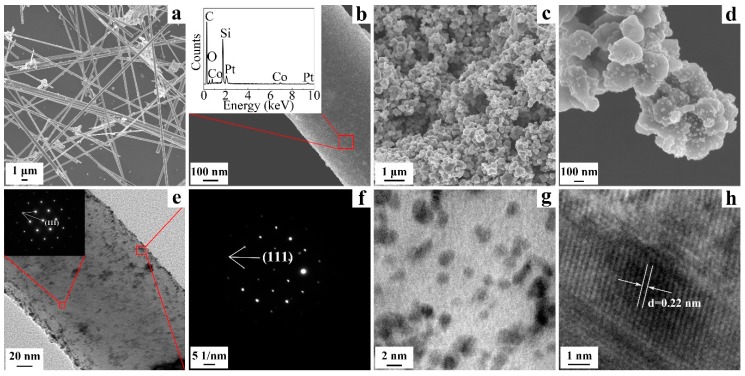
SEM images of Pt_3_Co-SiC nanowires (**a**,**b**); Pt_3_Co-SiC powders (**c**,**d**); TEM images of Pt_3_Co-SiC nanowires (**e**,**f**); Pt_3_Co-SiC powders (**g**,**h**).

The morphology and microstructure of the product are characterized using SEM and TEM analysis. The images of Pt_3_Co-SiC nanowires (Figure 3a,b) and powders (Figure 3c,d) at low and high magnification clearly disclosed that Pt_3_Co nanoparticles are homogeneously dispersed on the surface of SiC nanowires and powders, respectively. To further confirm the existence of Pt_3_Co nanoparticles, EDS is performed and the result is shown in Figure 3b. There are 5.11 wt % metal or metal alloy loaded according to the EDS data, which corresponds well to our expectation. TEM images of the Pt_3_Co-SiC nanowires are given in Figure 3e. In the inset of Figure 3e, the fast fourier transform (FFT) of the nanowire shows lattice reflections of cubic SiC (111). Figure 3f reveals that the FFT of the black nanoparticles corresponds to the lattice reflections of Pt_3_Co (111). The nanoparticles of 2–5 nm in size are uniformly dispersed on the surface of the SiC powders (Figure 3g). The HRTEM image of Pt_3_Co-SiC nanoparticles (Figure 3h) indicates two adjacent, well-defined lattice fringes with an interplanar distance of 0.22 nm, which is further indexed to the (111) plane of Pt_3_Co.

### 2.2. The Photoelectrochemical Property of SiC, Modified with Pt-Co Alloy

**Figure 4:**
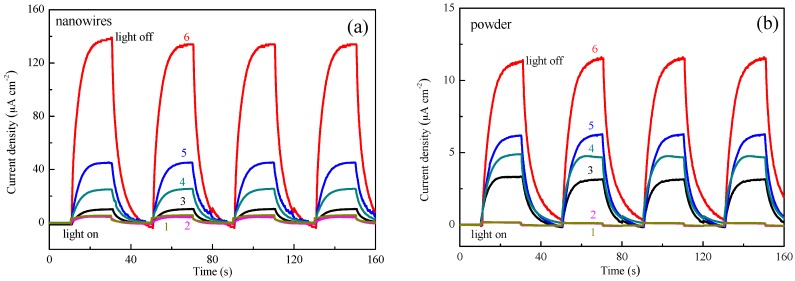
Current density of the different SiC nanowires photoelectrode: Pt_3_Co-SiC (6), PtCo-SiC (5), PtCo_3_-SiC (4), Pt-SiC (3), Co-SiC (2), SiC (1) at open circuit voltage in the dark and simulated solar light (**a**); different SiC powders photoelectrode: Pt_3_Co-SiC (6), PtCo-SiC (5), PtCo_3_-SiC (4), Pt-SiC (3), Co-SiC (2), SiC (1) at open circuit voltage in the dark and simulated solar light (**b**).

To assess the photoresponse and stability of the SiC modified with Pt-Co alloy photoelectrodes, current vs. time (I-t) curves are studied. The photoelectrode is investigated using switched light in a Na_2_S/Na_2_SO_3_ solution, at a constant potential, at open circuit voltage (−0.6 V). As shown in Figure 4, a sharp increase in photocurrent in the positive direction is observed upon illumination (light), which reverts to the initial state as soon as the illumination is turned off (dark). The photoresponse characteristics remain almost constant for several cycles of operation, indicating good photoreversibility and the stability of the present photoelectrodes. The different molar ratio of Pt-Co alloys loaded on SiC nanowires and powders are investigated. By comparison, the photogenerated current density for Pt_3_Co-SiC is the highest. Especially, the photogenerated current density is 140 µA·cm^−2^ for the Pt_3_Co-SiC nanowires under visible light irradiation (Figure 4a), which is about 10 times higher than the Pt_3_Co-SiC powders (Figure 4b). Compared with the SiC powders, the SiC nanowires exhibit higher photoelectrocatalytic activity. The main reasons for the better performance of the nanowires are as follows. On the one hand, nanowires possess better dispersity than that of nanoparticles. On the other hand, the nanoparticles have a high recombination of the photogenerated charges, according to the photoluminescence (PL) spectra.

### 2.3. Water Splitting for Hydrogen

**Figure 5:**
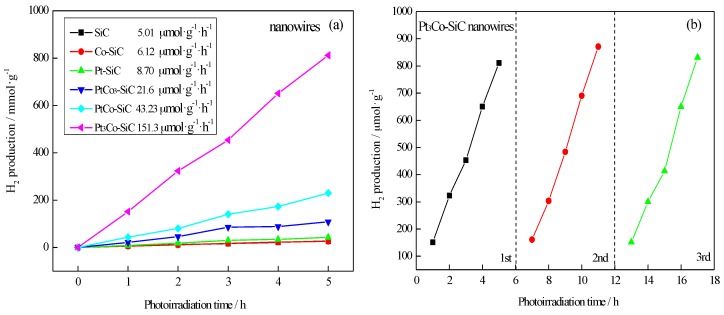
(**a**) Photocatalytic hydrogen evolution performance over: Pt_3_Co-SiC, PtCo-SiC, PtCo_3_-SiC, Pt-SiC, Co-SiC, SiC; (**b**) cyclic experiments of Pt_3_Co-SiC nanowire over water splitting.

The photocatalytic activities for H_2_ production of the SiC nanowires and the SiC nanowires modified with Pt-Co alloy were evaluated under visible light irradiation in aqueous suspensions with Na_2_S and Na_2_SO_3_ as sacrificial agents (electron donors). As shown in Figure 5a, the photocatalytic activities of the SiC nanowires with a different treatment were compared. It can be seen that the Pt-Co alloy significantly affects the photocatalytic activity. The photocatalytic H_2_ production rates of the treated samples from high to low are Pt_3_Co-SiC (151.3 μmol·h^−1^·g^−1^), PtCo-SiC (43.23 μmol·h^−1^·g^−1^), PtCo_3_-SiC (21.6 μmol·h^−1^·g^−1^), Pt-SiC (8.70 μmol·h^−1^·g^−1^), Co-SiC (6.12 μmol·h^−1^·g^−1^), and pure SiC (5.01 μmol·h^−1^·g^−1^). Stability tests for photocatalytic hydrogen production using the Pt_3_Co-SiC nanowires were also investigated by carrying out recycling reactions three times under visible light irradiation. No decrease in catalytic activity was observed in the recycling reactions, as shown in Figure 5b. The H_2_ production rate for the Pt_3_Co-SiC nanowires exceeds by over 30 times the pure SiC nanowires. These Pt_3_Co-SiC NWs also exhibit an enhanced activity for H_2_ production compared with recent work, such as the modified SiC nanowires [30], RGO/SiC [31], and the boron-doped SiC nanowires [32], SiC-PEDOT/PSS [33], as shown in Table 1. It confirms that noble metals-based alloys can maintain higher photocatalytic activity.

### 2.4. Photoresponse Mechanism

**Figure 6:**
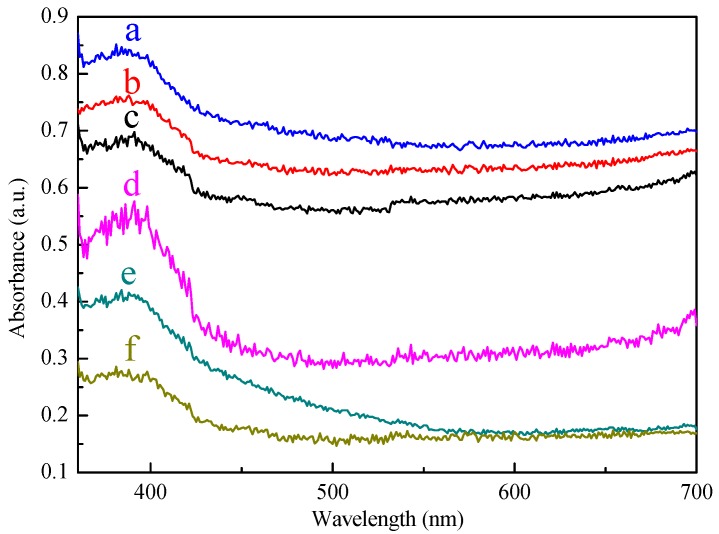
UV-Vis diffusion reflectance spectra of Pt_3_Co-SiC (**a**); PtCo-SiC (**b**); PtCo_3_-SiC (**c**); Pt-SiC (**d**); Co-SiC (**e**); SiC (**f**).

Figure 6 shows the UV-Vis absorption spectra of the SiC, the Pt-SiC, the Pt_3_Co-SiC, the PtCo-SiC, the PtCo_3_-SiC, and the Co-SiC samples. The maximum absorption wavelength of the catalyst can be achieved by extending the tangent of the critical fall section of the UV-Vis diffuse spectrum to the horizontal axis [34]. The maximum absorption wavelength of SiC is estimated to be about 464 nm, which indicates that SiC responds to visible light. After the introduction of the Pt and Co species, the maximum absorption wavelengths of Pt-SiC, Pt_3_Co-SiC, PtCo-SiC, PtCo_3_-SiC, and Co-SiC are approximately 515, 585, 573, 561, and 488 nm, respectively. The band gap of SiC calculated based on the Kubelka-Munk method is 2.42 eV, which accorded well with reports. The band gap of Pt-SiC, Pt_3_Co-SiC, PtCo-SiC, PtCo_3_-SiC, and Co-SiC are 2.17, 2.05, 2.08, 2.10, and 2.26 eV, respectively. It is quite clear that the existence of Pt-Co alloy reinforces the absorption wavelength of catalysts in the visible light region.

**Figure 7:**
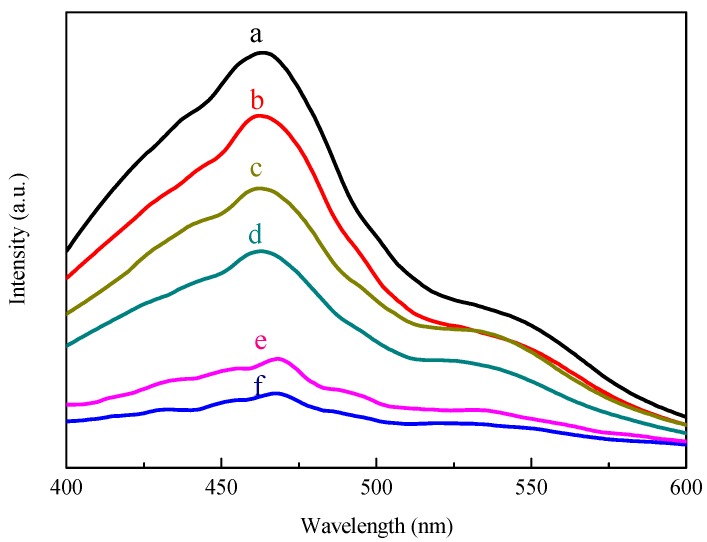
PL spectra of SiC (**a**); Co-SiC (**b**); Pt-SiC (**c**); PtCo_3_-SiC (**d**); PtCo-SiC (**e**); Pt_3_Co-SiC (**f**).

**Figure 8:**
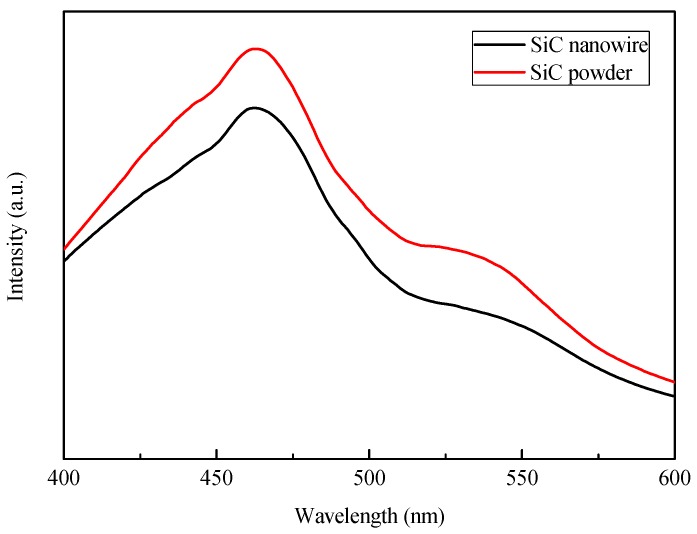
PL spectra of SiC powder and SiC nanowire.

As for the enhanced photoresponse mechanism for the PtCo_3_-SiC electrode, the recombination and transportation of the photogenerated charges were further investigated by the photoluminescence (PL) spectra. Figure 7 shows the PL spectra of the SiC, the Pt-SiC, the Pt_3_Co-SiC, the PtCo-SiC, the PtCo_3_-SiC, and the Co-SiC nanowire samples. PL measurement of the catalysts with an excitation wavelength at 340 nm was carried out at room temperature, in which the peak centred at 468 nm is ascribed to the bandgap recombination of 3C-SiC [35]. After the introduction of the Pt and Co species, the PL intensity of Co-SiC, Pt-SiC, PtCo_3_-SiC, PtCo-SiC, and Pt_3_Co-SiC decreases gradually, and the maximum decrease in PL intensity at around 468 nm implies the highest separation rate of photogenerated electron-hole pairs. It is quite clear that the existence of the Pt-Co alloy reinforces the separation of photogenerated electron-hole pairs, and that Pt_3_Co-SiC is the best. The PL spectra of the SiC powder (red line) and SiC nanowire (black line) are shown in Figure 8. It is obvious that the SiC nanowires exhibit a better performance in the separation of electron-hole pairs.

**Figure 9:**
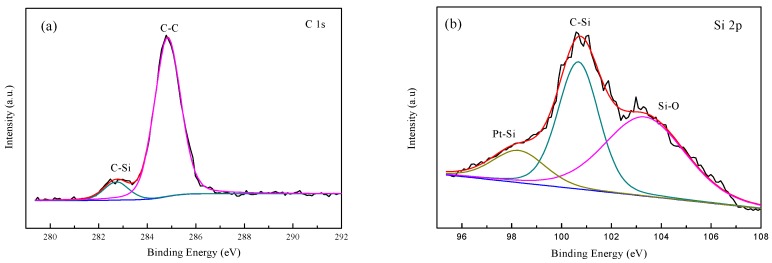

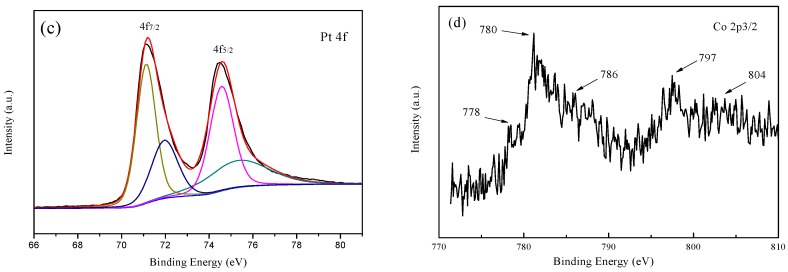
XPS spectra of the Pt_3_Co–SiC nanowires: (**a**) C 1s; (**b**) Si 2p; (**c**) Pt 4f; (**d**) Co 2p3/2.

In addition, XPS is applied to obtain the surface elemental compositions and valence states of the prepared samples. Figure 9 present the XPS spectra of the Pt_3_Co-SiC nanowires. The survey XPS spectrum indicates that the main elements on the surface of the products are C, Si, and a small amount of Pt and Co. Figure 9a shows the C 1s XPS spectrum of the Pt_3_Co-SiC nanowires, in which two XPS peaks are observed at approximately 282.6 eV and 284.8 eV; the characteristic peaks of C–Si and C–C bond, respectively. Figure 9b gives the spectrum of the Si–C and Si–O peaks in the samples, which are located at 100.8 eV and 103.2 eV, respectively. Significantly, the peak centered at 98.3 eV, corresponding to the Pt–Si bond [36], is presented in Figure 9b. The reason for the formation of the Pt–Si bond is that reduced Pt ions deposit around the Si atoms and then grow into particles, rather than the displacement reaction that always occurs at a high temperature [37]. Due to the formation of the Pt–Si bond, an excellent electron transport channel is established. This effect accelerates the excited electrons transfer from Si to the Pt–Co surface, which is beneficial for decreasing the electron-hole recombination [38]. Figure 9c presents the Pt 4f spectrum of the as-prepared sample; two peaks are located at 71.2 eV and 74.8 eV, which are ascribed to the Pt° 4f 7/2 and Pt° 4f 5/2 binding energies. The peaks at 72.4 eV and 75.6 eV are attributed to Pt (II) [39]. The Co 2p spectrum (Figure 9d) is characterized by two major peaks of Co 2p3/2 and Co 2p1/2 at 780 eV and 797 eV. Moreover, satellites centered at 786 eV and 804 eV also confirm the formation of Co (II) [40]. The peak at 778 eV exhibits the presence of Co (0), and its strength is weak because Co is a more oxophilic metal compared with Pt and is easier to show the effect of oxidation [41]. The valence state of Co after the photocatalytic reaction is similar to before. The actual composition of the expected Pt_x_Co_y_ alloys can be obtained by the XPS spectra. The actual composition ratios of Pt_3_Co, PtCo and PtCo_3_ are Pt_2.89_Co, Pt_0.94_Co_1_, PtCo_2.86_, respectively.

**Figure 10:**
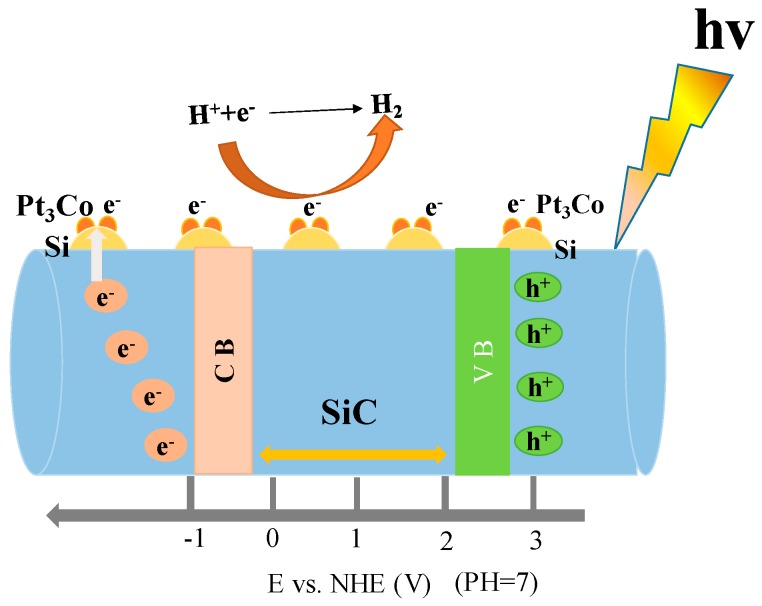
Schematic diagram of photocatalytic mechanism for H_2_ production over Pt_3_Co-SiC.

Based on the above mentioned experimental results, a possible mechanism of the photocatalytic activity enhancement of the alloys-SiC is proposed (as shown in Figure 10). Cubic SiC stimulated by visible-light and electron-hole pairs is generated under visible light irradiation. Without any co-catalyst, the Si atom acts as a reductive reaction site. When the nano-Pt_3_Co alloy is deposited on the SiC surface, Pt_3_Co nanoparticles act as a reductive reaction site instead of Si. Through the particularly effective Pt-Si bond, an excellent electron transport channel is established. Meanwhile the “synergistic effect” of Pt and Co changes the Fermi energy level of the Pt_3_Co alloys, and then the electrons’ trapping ability is enhanced for the Pt_3_Co alloy in comparison to Pt [42]. The specific band edge positions of the CB and valance band (VB) of SiC can be estimated by the following equation [43].

E_CB_ = x − E^e^ −1/2E_g_ (1)

where E_CB_ is the CB edge potential. x is the electronegativity of the semiconductor, expressed as the geometric mean of the absolute electronegativity of the constituent atoms, which is defined as the arithmetic mean of the atomic electron affinity and the first ionization energy. E^e^ is the energy of free electrons on the hydrogen scale ca., while 4.5 eV. E_g_ is the band gap of the semiconductor. The calculated CB and VB of SiC are −0.24 and 2.18 eV. Moreover, in the Pt_3_Co-SiC system, the photo-generated electrons in the CB of the SiC transfer to Pt_3_Co co-catalysts via contacting interfaces, giving the conduction band electrons higher mobility and promoting the separation of the electron–hole pairs. The holes in the VB of SiC are consumed by Na_2_S/Na_2_SO_3_ sacrificial regents. It can cut down the recombination of the photogenerated electrons and holes. Subsequently, the electrons in Pt_3_Co nanoparticles transfer to the reduction of H^+^ to H_2_. Therefore, the Pt_3_Co-SiC samples exhibit superior activity for the hydrogen production.

## 3. Experimental Section

### 3.1. Materials

Silica (SiO_2_ > 99%), tetraethyl orthosilicate (Si(OC_2_H_5_)_4_, TEOS), carbon black (C ≥ 99.5%), chloroplatinic acid (H_2_PtCl_6_·6H_2_O), cobalt nitrate (Co(NO_3_)_2_·6H_2_O), hydrochloric acid (Hcl), and hydrofluoric acid (HF) were provided by Sinopharm Chemical Reagent Beijing Co., Ltd. (SCRB) (Beijing, China). Argon with a purity of 99.99% was supplied by Haipu Gas Co., Ltd. (Beijing, China). Indium-Tin Oxide (ITO) conductive film glasses with a size of 1 cm × 1 cm were provided by Shenzhen Jingweite Technology Co., Ltd. (Shenzhen, China).

### 3.2. Preparation of the SiC Nanowires and Powders

The SiC nanowires were prepared as follows: Silica and carbon black with the mass ratio of Si:C = 1:1 were mixed. The powder that was put in a ceramic boat was placed at the hot zone of a furnace. Then, the furnace was put under vacuum and argon was leaded into at a constant gas flow rate of 50 sccm with a pressure of 1 atm. The furnace was heated to 1500 °C and held for 2 h. It was cooled to 800 °C at a rate of 3 °C·min^−1^ in flowing argon and then cooled naturally to room temperature in air. Finally, the nanowires were calcined at 700 °C for 3 h in the air to eliminate the unreacted carbon and washed with 10% HF for 1 h to remove the residual silica [44].

SiC powders were prepared through the sol-gel method. The process was as follows: the sol mixture was prepared using TEOS and carbon black, with the mass ratio of Si:C = 1:1 as the silicon source and the carbon source, respectively. Distilled water and ethanol were used as solvents. TEOS, ethanol, carbon black, and water were mixed under stirring and the solution pH was adjusted to 4.0 using HCl. The prepared sol was dried at 80 °C to obtain gel. The dry gel was put into a graphite crucible and was heated in a furnace at 1500 °C for 4 h in flowing argon. It was cooled naturally to room temperature in air and the powder was obtained. At last, the powder was dealt with in the same way as that of the nanowires to eliminate the unreacted carbon and residual silica.

### 3.3. Synthesis of the Pt-Co-SiC Electrode

The synthesis procedure of SiC modified with the Pt-Co alloy was as follows: firstly, solution A was prepared using 60 mL ethylene glycol and 15 mL deionized water with the pH value of 11.0 adjusted by NaOH. Then, H_2_PtCl_6_ and Co(NO_3_)_2_·6H_2_O with a Pt/Co molar ratio of 1:0, 3:1, 1:1, 1:3, 0:1 were dissolved in solution A and magnetically stirred for 10 min to form a homogeneous and light green solution. Subsequently, 10 mg SiC nanowires or powders were put into the above solution and stirred for 30 min at high speed and ultrasonication for 30 min to ensure SiC disperse homogeneously. The obtained solution was poured into a 100 mL Teflon-lined stainless steel autoclave and heated to 200 °C for 10 h. Finally, the black precipitate was separated by centrifigation and washed using deionized water, acetone, and alcohol several times and dried at 80 °C in vacuum.

The working electrodes were prepared by dropping Pt-Co-SiC catalysts onto ITO glass. During the experiment, 1 mg of the catalysts were ultrasonically dispersed in 0.4 mL of 0.05 wt % of the Nafion solution to form homogeneous suspension [45]. 0.02 mL of the above mentioned suspension (containing 0.05 mg of the catalyst) was dip-coated onto a 1 cm × 1 cm ITO glass electrode. Finally, the electrodes were dried in a vacuum oven at 70 °C overnight to evaporate all of the ethanol.

### 3.4. Characterization

The surface morphology was characterized on a Hitachi SU8020 scanning electron microscope (SEM, Hitachi Ltd., Tokyo, Japan) and transmission electron microscopy (TEM JEOL JEM-2010). The phase of the product was carried out using X-ray diffraction (XRD, TTRIII, Rigaku, Bruker, Karlsruhe, Germany with Cu Kα radiation). The photoluminescence (PL) spectra were investigated on a Hitachi F-4500 fluorescence spectrometer. X-ray photoelectron spectroscopy (XPS) was performed on a VG Multilab 2009 system (Manchester, UK).

### 3.5. Photoelectrochemical Measurements

The photoelectrochemical analyses were carried out on a CHI 760C (Chenhua Ltd., Shanghai, China) electrochemical workstation by a three-electrode configuration. The prepared Pt_3_Co-SiC electrodes, Pt foil, and saturated calomel electrode acted as the working, counter, and reference electrodes, respectively. The electrolyte was a 0.25 mol·L^−1^ Na_2_S and 0.35 mol·L^−1^ Na_2_SO_3_ solution. The open circuit voltage is measured in the dark. The photocurrent density with time (I-t curve) was performed at the measured circuit voltage under visible light irradiation. The radiation source was obtained by a 300 W xenon arc lamp.

### 3.6. Photocatalytic Tests

Photocatalytic hydrogen production experiments were carried out in an air-free closed gas circulation system reaction cell made of quartz. The total cylindrical volume of the cell was 200 mL. An optically polished piece of quartz glass was fused on top of the cell to minimize light scattering. Hydrogen evolution was detected using a gas chromatograph (GC-3240, Yuanhong technology Co., Ltd., Beijing, China, TCD, Ar carrier), which was connected to a gascirculation line. Argon, with a flow rate of 100 mL·min^−1^, was used as a carrier gas, and was passed through the quartz glass cell. In a typical photocatalytic experiment, 0.2 g of the prepared photocatalyst was dispersed with constant stirring in a 200 mL mixed solution of Na_2_S (5 mL, 0.1 mol·L^−1^), Na_2_SO_3_ (5 mL, 0.04 mol·L^−1^), and distilled water (190 mL). Na_2_S, due to its more negative oxidation potential, was often used as a sacrificial reagent [9,43,46,47]. The reaction was initiated by irradiation with a 300 W xenon lamp fitted with a cutoff filter (λ > 420 nm). The light intensity employed was 100 mW·cm^−2^. The whole system, including the photocatalyst, was flushed with Ar at 100 mL·min^−1^ for 1 h to remove any trace of air (including nitrogen and oxygen) before any photocatalytic reaction was carried out. During the process, agitation of the solution ensured uniform irradiation of the suspension. A 0.4 mL sample of the generated gas was collected intermittently through the septum, and the hydrogen content was analyzed by a gas chromatograph (GC-14C, Shimadzu, Tokyo, Japan, TCD, nitrogen as a carrier gas, and a 5 Å molecular sieve column). All glassware was rigorously cleaned and carefully rinsed with distilled water prior to use.

## 4. Conclusions

The Pt-Co-SiC catalyst was prepared via an ethylene glycol reduction method. The Pt-Co alloy nanoparticles were uniformly loaded on the surface of SiC. The maximum photocatalytic activity was achieved when the ratio of Pt:Co was 3:1. As for the enhanced photocatalytic mechanism, Pt_3_Co nanoparticles acted as a reductive reaction site by forming the particular effective Pt-Si bond, and thus an excellent electron transport channel was established. In the photocatalytic process, the Pt_3_Co alloy nanoparticles could capture electrons from SiC to the Pt_3_Co alloy. It could decrease the recombination of the photogenerated electron-hole pairs.

## Figures and Tables

**Table 1:** Comparison of H_2_ production of semiconductor photocatalysts reported in the literature with our work.

Material	Morphology	H_2_ Production (μmol·h^−1^·g^−1^)	Year/Reference
Modified SiC	nanowires	2.68	2012/[30]
RGO/SiC	powder	42.4	2013/[31]
SiC	fibre	67.5	2015/[33]
SiC-PEDOT/PSS	fibre	100.7	2015/[33]
B-SiC	nanowires	108.4	2015/[32]
Pt_3_Co-SiC	nanowires	151.3	This work
